# Hemiplegic Migraine: An Imitator of Cerebrovascular Disease

**DOI:** 10.7759/cureus.99556

**Published:** 2025-12-18

**Authors:** Aria Kana, Sontosh Reddy, John Fox, Omar Kawam, Ahmed Kawamj

**Affiliations:** 1 Biology, Hunter College High School, New York, USA; 2 Internal Medicine, New York Medical College/St. Michaels Medical Center, Newark, USA; 3 Internal Medicine, Robert Wood Johnson Medical School, New Brunswick, USA

**Keywords:** cochlear implant, dysarthria, hemiplegic migraine, migraine, stroke, systemic lupus erythematosus

## Abstract

Hemiplegic migraine is a rare subtype of migraine with aura characterized by reversible unilateral motor weakness, often accompanied by transient visual, sensory, or speech disturbances. Because the motor deficits frequently mimic acute cerebrovascular events, patients often undergo urgent stroke evaluations, creating significant diagnostic uncertainty. We present the case of a 72-year-old woman with a clinical stroke with negative MRI imaging 10 months prior, who presented to the emergency department with stroke-like symptoms for the past three days and a National Institutes of Health Stroke Scale (NIHSS) score of 5. Head CT and CT angiography confirmed no hemorrhage. Subsequent work-up revealed no acute or remote ischemia or infarction. This case highlights the diagnostic challenge posed by the clinical overlap between hemiplegic migraine and ischemic stroke. Although structural imaging is often normal in hemiplegic migraine, early presentation before symptom resolution can prompt unnecessary therapy. The case highlights the importance of thorough history-taking for early recognition and appropriate management of hemiplegic migraine.

## Introduction

Migraines can present with an aura that localizes to the cerebral cortex or brainstem and may involve visual disturbances, sensory loss, dysphagia, or motor involvement. Hemiplegic migraine is a rare subtype of migraine with aura characterized by transient motor weakness or hemiplegia during attacks. In sporadic hemiplegic migraine, these neurologic deficits arise in the absence of a family history [1]. Patients frequently experience severe headache accompanied by photophobia, numbness, tingling, paresthesias, dysarthria, and temporary motor impairment that can persist from minutes to several days [2]. Motor manifestations, including hemiparesis or hemiplegia (80-99%), ataxia and nystagmus (30-79%), and dysphagia (5-29%), can closely resemble acute ischemic stroke, contributing to a high rate of initial misdiagnosis [3]. Because the underlying pathophysiology and evidence-based management of hemiplegic migraine remain poorly understood, timely recognition relies heavily on clinical judgment, detailed history-taking, and exclusion of secondary causes, as treatment delays or inappropriate interventions may adversely affect outcomes.

Although hemiplegic migraine is already an uncommon clinical entity, late-onset presentations are exceptionally rare, with most cases occurring in childhood or early adulthood. When hemiplegic migraine first emerges in older adults, diagnostic challenges are amplified. Elderly patients often possess multiple vascular risk factors, such as hypertension, atrial fibrillation, diabetes, or prior cerebrovascular events, as well as autoimmune comorbidities, all of which increase the pre-test probability for transient ischemic attack (TIA) or acute ischemic stroke [4]. As a result, hemiplegic migraine is typically not considered early in the differential diagnosis, despite the potential for fully reversible neurologic deficits.

This overlap in symptomatology creates a critical diagnostic dilemma. Sudden-onset unilateral weakness, sensory disturbance, dysarthria, and visual changes are hallmark features shared by hemiplegic migraine, TIA, and stroke. The absence of a clear preceding headache, the variability of aura progression, and the fluctuating nature of deficits can further obscure the clinical picture. Distinguishing these entities is essential, as the management of acute stroke, including thrombolysis or anticoagulation, carries significant risks if administered to a patient with hemiplegic migraine, while failure to recognize an evolving cerebrovascular event may delay life-saving treatment.

Given these challenges, expanding clinical awareness of late-onset hemiplegic migraine is crucial. More explicit recognition of its mimicry of cerebrovascular disease can help reduce misdiagnosis, guide appropriate diagnostic evaluation, and improve outcomes in older adults who present with transient neurologic symptoms.

## Case presentation

A 72-year-old woman with a past medical history significant for systemic lupus erythematosus (SLE), rheumatoid arthritis (RA), migraines (on topiramate 50 mg twice a day, and a prior clinical diagnosis of stroke in December 2024 with negative brain MRI, presented with complaints of new-onset left-sided weakness, left eye ptosis, occipital headache, and slurred speech. Her prior stroke evaluation included a transthoracic echocardiogram and cardiac CT, both of which were unremarkable, and an implantable loop recorder that showed no arrhythmic events. She had also undergone left cochlear implant insertion.

The patient reported that since her admission in December 2024, she has experienced intermittent episodes of left-sided weakness associated with headache. These episodes typically occurred several times per month, lasting one to two days before spontaneously resolving. The current episode began in October 2025 and progressively worsened, with the patient describing feeling “very weak” two days later.

**Figure 1:**
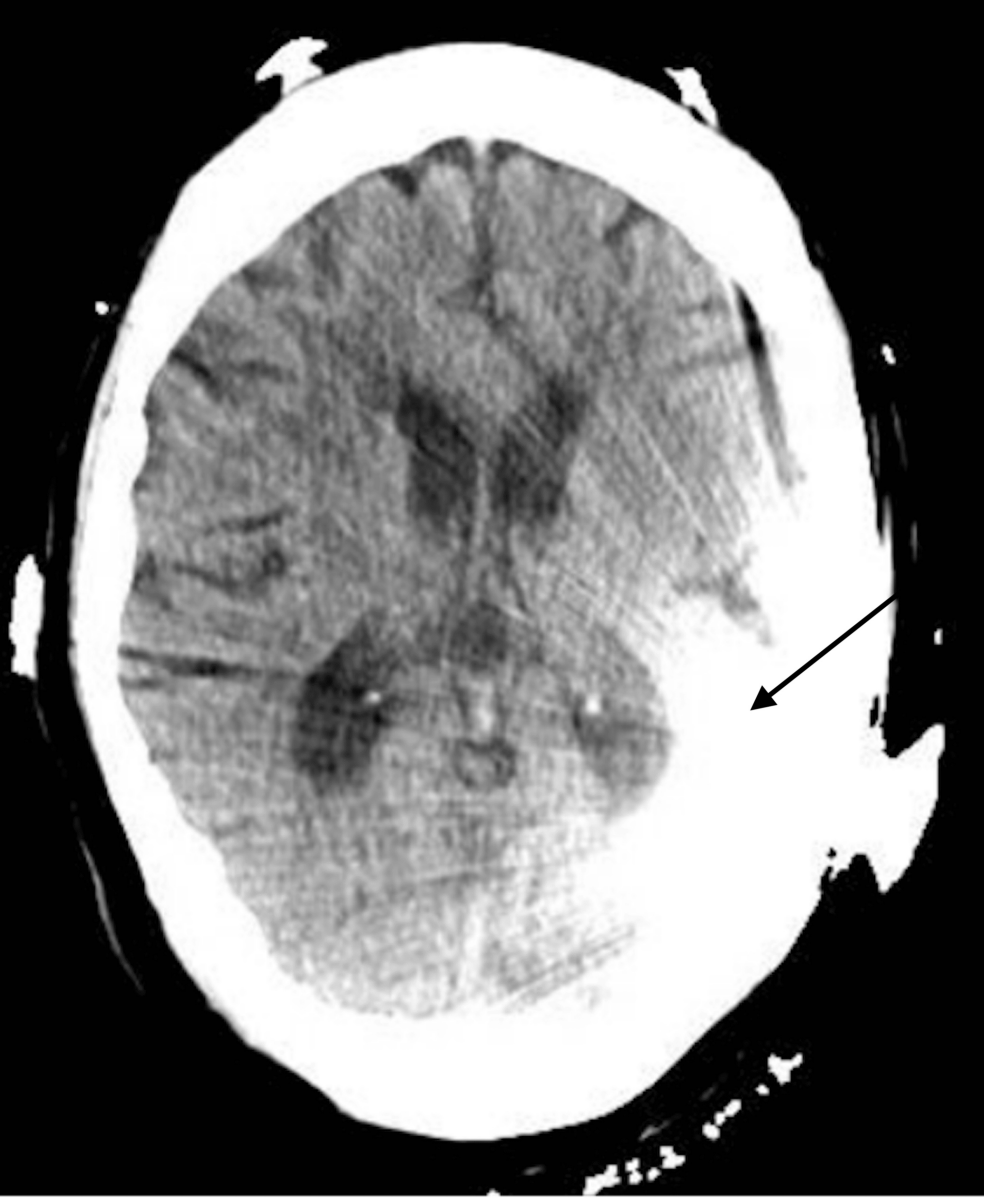
CT Head showing an artifact with cochlear implant

**Figure 2:**
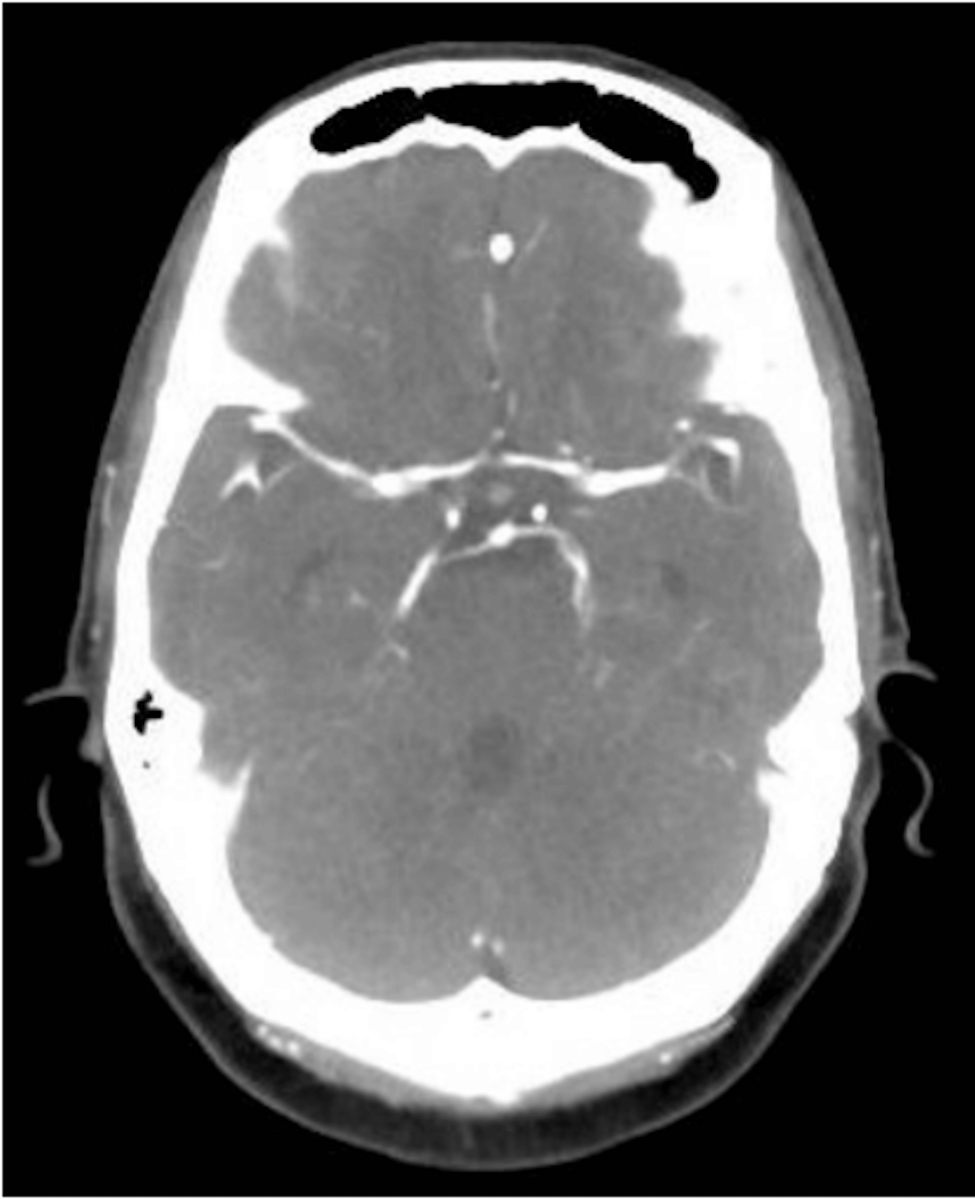
CT angiography Brain with no significant findings

On arrival to the emergency department, her blood pressure was in the 170s/110s mmHg. A non-contrast CT head (Figure 1) and CT angiography of the head and neck (Figure 2) were obtained and were unremarkable, though partially limited by artifact from the left cochlear implant. Neurological examination revealed a National Institutes of Health Stroke Scale (NIHSS) score of 5, with findings including left nasolabial fold flattening, left upper extremity weakness (4-/5 with drift not hitting the bed), left lower extremity weakness (3/5 briefly antigravity), and decreased sensation involving the left face, arm, and leg.

The patient reported adherence to aspirin and statin therapy for secondary stroke prevention. She was admitted to the stroke service with repeat imaging being unremarkable. Labs were unremarkable (Table 1). An MRI was unable to be obtained due to the cochlear implant, which represents a major limitation in definitively excluding cerebral ischemia. However, the clinical criteria for hemiplegic migraine described by the International Classification of Headache Disorders were met. Blood cultures and infectious workup were negative for HIV, herpes simplex virus (HSV), and varicella-zoster virus (VZV). Video EEG monitoring for 48 hours revealed no seizure activity. Symptoms improved over the course of the patient’s hospitalization, and she was discharged on an increased dose of topiramate 100 mg twice a day.

**Table 1:** Laboratory test results

Test	Patient Value	Reference Range
White Blood Cells	4.5 10*3/uL	3.80-10.50 10*3/uL
Hemoglobin	12.9 g/dL	11.5-15.5 g/dL
Platelets	167 10*3/uL	150-400 10*3/uL
Sodium	143 mmol/L	135-145 mmol/L
Potassium	3.6 mmol/L	3.5-5.3 mmol/L
Blood Urea Nitrogen	21 mg/dL	7-23 mg/dL
Creatinine	0.9 mg/dL	0.5-1.30 mg/dL
Calcium	9.2 mg/dL	8.5-10.5 mg/dL
Alkaline Phosphatase	126U/L	40-120 U/L
Aspartate Aminotransferase (AST)	38 U/L	10-40 U/L
Alanine Aminotransferase (ALT)	27U/L	10-45 U/L

## Discussion

Headache disorders are among the most prevalent neurological conditions worldwide. The World Health Organization estimates that 50-75% of adults aged 18-65 years have experienced a headache in the past year, and up to 30% of these individuals have reported migraine episodes [5]. Migraines are characterized by recurrent, paroxysmal headaches that may occur with or without aura. The underlying mechanism is thought to involve cortical spreading depression, which activates the trigeminal nucleus caudalis and is believed to originate in the occipital cortex before spreading anteriorly [6]. Vasogenic leakage from leptomeningeal vessels may stimulate the trigeminovascular system, contributing to the aura experienced in some patients [7].

Hemiplegic migraine is a rare subtype of migraine with aura that was first described by Clarke in 1910 [8]. The reported prevalence is approximately 0.01% [9]. Hemiplegic migraine is characterized by transient hemiparesis or hemiplegia occurring during an episode of migraine, with weakness that may resolve before the headache or persist for several days to months. The aura often includes visual, sensory, or speech disturbances, and the attacks are frequently triggered by factors such as stress, sleep disturbances, bright lights, or intense emotional stimuli.

Based on family history, hemiplegic migraine can be classified as either familial or sporadic. Familial hemiplegic migraine is diagnosed when a first- or second-degree relative experiences similar attacks, while sporadic hemiplegic migraine is diagnosed in the absence of such a history [10]. The familial subtype typically presents earlier in life, often in the first or second decade, and the frequency of attacks tends to decrease with age. In contrast, the sporadic subtype tends to present later in adulthood, as observed in our patient.

The diagnostic criteria for hemiplegic migraine, as defined by the International Classification of Headache Disorders, 3rd edition (ICHD-3), include recurrent episodes of migraine with fully reversible motor weakness and other aura symptoms (visual, sensory, or language-related) [11]. These symptoms typically develop gradually, last five minutes to an hour, and are usually followed by headache within one hour.

Genetic studies have identified several mutations associated with familial hemiplegic migraine, including *CACNA1A *(FHM1), *ATP1A2* (FHM2), and *SCN1A* (FHM3) [12]. These genes are transmitted in an autosomal dominant pattern with variable penetrance. Approximately 10-20% of sporadic hemiplegic migraine patients may carry one of these mutations, particularly *ATP1A2*. However, routine genetic testing is not required for diagnosis and is reserved for atypical or severe cases. A Danish population-based study found that only 14% of familial hemiplegic migraine patients had mutations in these genes, suggesting additional unidentified genetic factors [13]. Mutations in *PRRT2*, associated with benign familial infantile epilepsy, have also been implicated in hemiplegic migraine and may warrant seizure prophylaxis in addition to migraine prevention [14].

Given its overlapping presentation with cerebrovascular events, hemiplegic migraine can easily be mistaken for ischemic stroke or TIA. In our patient, neuroimaging and laboratory investigations ruled out structural, infectious, and vascular causes, and the complete reversibility of neurological symptoms supported the diagnosis of sporadic hemiplegic migraine.

Management of hemiplegic migraine parallels that of migraine with aura, although standardized guidelines are lacking due to the rarity of the condition. Acute management focuses on pain control using nonsteroidal anti-inflammatory drugs (NSAIDs) and non-narcotic analgesics. Triptans and ergot derivatives are contraindicated because of their vasoconstrictive effects and potential risk of inducing ischemia [15]. Preventive therapy is considered for patients with frequent or debilitating episodes. Commonly used agents include flunarizine, verapamil, lamotrigine, acetazolamide, topiramate, valproic acid, and amitriptyline [16]. While evidence from randomized trials is unavailable, case series suggest potential benefit.

In our case, the patient was initially treated with topiramate for prophylaxis but discontinued therapy due to financial constraints, leading to recurrent attacks. This highlights the importance of consistent follow-up, accessibility of medication, and patient education in preventing recurrence and improving long-term outcomes in hemiplegic migraine.

## Conclusions

Hemiplegic migraine is a rare but important stroke mimic that requires cautious clinical evaluation and exclusion of secondary causes. Its presentation with transient hemiparesis can lead to significant diagnostic uncertainty, often prompting extensive neuroimaging and laboratory workup. Recognition of its characteristic reversible motor aura and adherence to ICHD-3 diagnostic criteria are essential to avoid unnecessary interventions and to guide appropriate management. Early diagnosis, patient education, as well as adherence to prophylactic therapy can reduce recurrence and improve quality of life. This case highlights the importance of maintaining a broad differential diagnosis in patients presenting with acute focal neurological deficits, especially in the absence of radiologic evidence of ischemia.
